# Enhancing and quantifying spatial homogeneity in monolayer WS_2_

**DOI:** 10.1038/s41598-021-94263-9

**Published:** 2021-07-21

**Authors:** Yameng Cao, Sebastian Wood, Filipe Richheimer, J. Blakesley, Robert J. Young, Fernando A. Castro

**Affiliations:** 1 National Physical Laboratory, Hampton Road, Teddington, TW11, 0LW UK; 2 Department of Physics, Lancaster University, Lancaster, LA1 4YB UK; 3 Advanced Technology Institute, University of Surrey, Guildford, GU2 7XH Surrey UK

**Keywords:** Two-dimensional materials, Imaging techniques

## Abstract

Controlling the radiative properties of monolayer transition metal dichalcogenides is key to the development of atomically thin optoelectronic devices applicable to a wide range of industries. A common problem for exfoliated materials is the inherent disorder causing spatially varying nonradiative losses and therefore inhomogeneity. Here we demonstrate a five-fold reduction in the spatial inhomogeneity in monolayer WS_2_, resulting in enhanced overall photoluminescence emission and quality of WS_2_ flakes, by using an ambient-compatible laser illumination process. We propose a method to quantify spatial uniformity using statistics of spectral photoluminescence mapping. Analysis of the dynamic spectral changes shows that the enhancement is due to a spatially sensitive reduction of the charged exciton spectral weighting. The methods presented here are based on widely adopted instrumentation. They can be easily automated, making them ideal candidates for quality assessment of transition metal dichalcogenide materials, both in the laboratory and industrial environments.

## Introduction

Recent progress in wafer-scale processing of two-dimensional (2D) transition metal dichalcogenides (TMDs)^1–6^ is driving interest in high-quality, large-area films for optoelectronic applications^3,4,7,8^. Large-area synthesis of 2D materials has advanced on numerous fronts, including chemical vapor deposition^9^, atomic layer deposition^10^, Van der Waals epitaxy^11^, and liquid phase methods^12^. However, insufficient control of lattice defects^8,13–16^, the dielectric environment^17^, and TMD-metal interface quality^18,19^ continues to limit the performance and reproducibility of fabricated devices^20^. These deficiencies typically manifest as spatial and spectral inhomogeneity, accompanied by low photoluminescence yield. Progress towards large-scale uptake will require techniques to quantify and to improve this material homogeneity.

Amongst methods to improve the homogeneity of exfoliated TMDs (and indeed graphene), the most well-known are hexagonal boron nitride (h-BN) encapsulation and flake suspension^21^. These transfer-based techniques have successfully led to demonstrations of high performance TMD-devices. However, the yield of these transfer techniques is sensitive to the substrate, adhesion, temperature, contamination, flake sizes and flake damage, limiting scalability and repeatability. Furthermore, encapsulation passivates the chemically and environmentally sensitive surface of 2D materials, preventing them from being used as sensors. Molecular superacid treatment is also useful to enhance homogeneity^22^, though its activation procedure is still being debated^23,24^. Clearly, there is a need for a non-contact, repeatable and scalable technique to enhance homogeneity that can feasibly be automated for integration into production lines to accelerate high quality manufacturing and open new market opportunities.

This paper presents a convenient method to improve the spatial and spectral homogeneity of exfoliated monolayer WS_2_, by using a simple laser treatment process that can operate at ambient conditions and be easily automated. Controlled laser-illumination improves the spatial homogeneity in the spectral characteristics of PL, which leads to increased PL efficiency^25^. The efficiency of photoluminescence (PL) and the form of the PL spectrum are very sensitive to both the electronic quality of TMD materials^22^ and the type of substrate can affect the formation of excitons and the optical bandgap via background doping^26^. Polydimethylsiloxane (PDMS) substrates^27,28^ have been shown to exhibit a low density of charge defects and therefore little charge transfer at the TMD/substrate interface (much akin to single h-BN flakes), and are very simple to fabricate. This makes PDMS substrates an attractive and practical platform for studying the exciton dynamics in single layers, where substrate interactions (e.g. charge-transfer or strain with SiO_2_/Si substrates) are minimal. Moreover, an effective spatial homogeneity study requires spatial sampling over a sufficient area, which is only possible on large area flakes. As an elastomer, PDMS effectively relaxes the strain in the monolayer, enabling routine and simple exfoliation of large area flakes suitable for studying spatial homogeneity^29^. In contrast, the process of laminating equivalently large flakes on SiO_2_/Si, sapphire, or indeed h-BN substrates typically requires an intermediate processing step, e.g. physical transfer^30^ or ultrasonication on metallic substrate first^31^, both of which lead to the formation of more defects that add complexity to the analysis. Using spatially resolved photoluminescence spectroscopy to characterise monolayer WS_2_ exfoliated onto PDMS stamps, we demonstrate a statistical analysis approach to quantify spatial homogeneity and therefore compare material quality. We expand on previous studies that applied laser annealing^32^ by demonstrating that it is possible to do so under purely ambient conditions, without having to prepare special substrates. Modelling of the time-series PL images suggests that spectral inhomogeneities are potentially coupled to other factors of flake inhomogeneity. The measurements and modelling presented here aim to complement previous reports^33,34^ that elucidate possible mechanisms of laser illumination induced spectral changes on monolayer TMDs.

## Results and discussion

### WS_2_ monolayer characterisation

**Figure 1:**
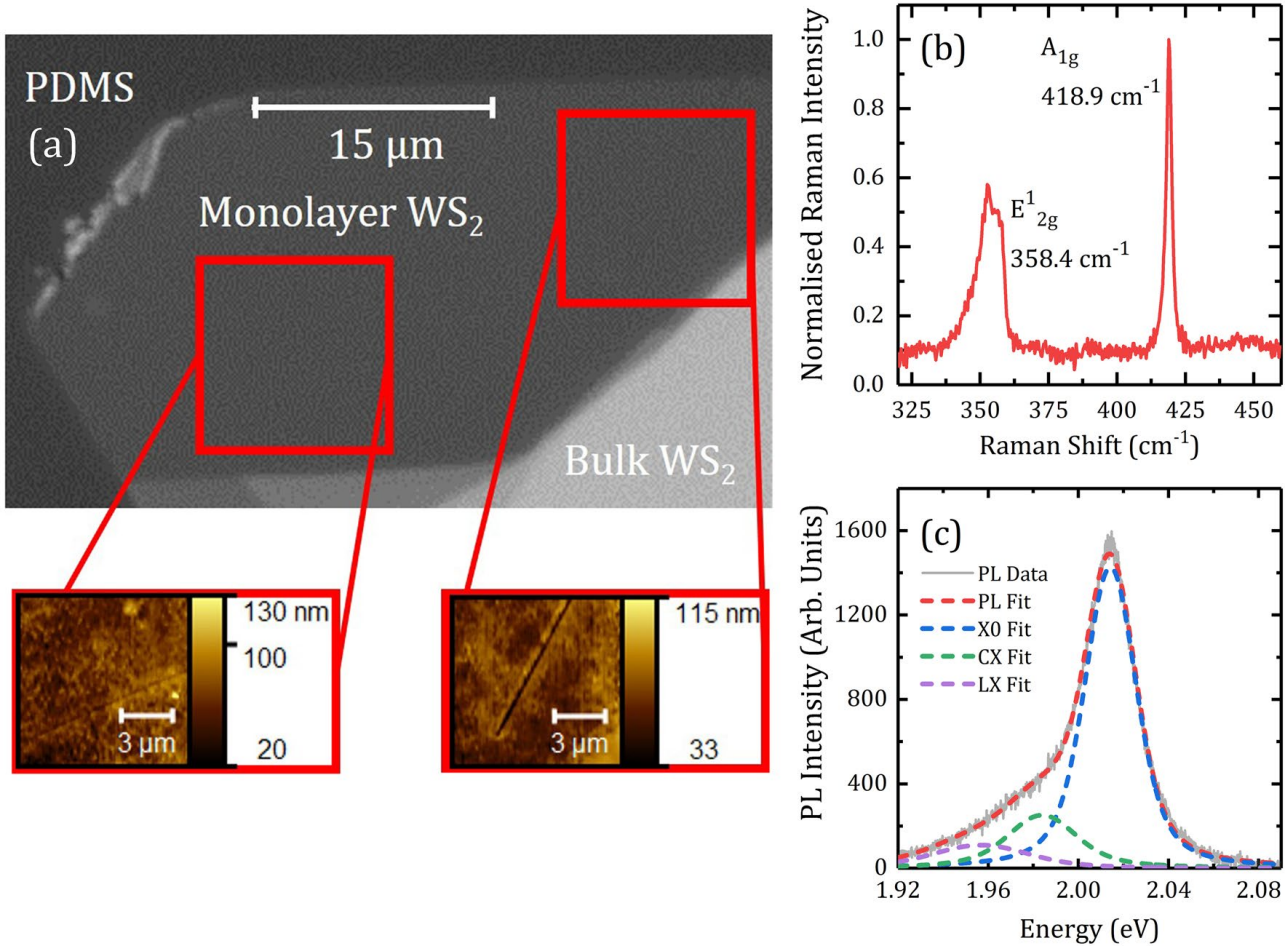
(**a**) Optical image of a WS_2_ monolayer flake obtained using dry exfoliation onto PDMS, AFM microscopy reveals crack defects, highlighted in red boxes. (**b**) A typical confocal Raman spectrum for the flake in (**a**), taken at room temperature with $633 \text{nm}$ excitation. (**c**) A typical PL spectrum, taken at 532 nm, at room temperature showing spectral components extracted using a Voigt model with fitted peak energies: ${E}_{X0}\approx 2.01 \text{eV}, {E}_{CX}\approx 1.98 \text{eV}, {E}_{LX}\approx 1.95 \text{eV}$, coefficient of determination for this fit is *R*^*2*^ = 0.996. More details about fitting can be found in the supplementary materials.

Monolayer WS_2_ flakes were prepared on PDMS stamps using ‘scotch tape’ exfoliation. Figure 1a shows an atomic force microscopy (AFM) topography image along with confocal Raman and PL spectra recorded at one position for one of the WS_2_ flakes studied in this paper. Soft polymers like PDMS are challenging for AFM and topographic height information in can have large uncertainties, due to the large elastic mismatch, between the WS_2_ and the PDMS substrate^35,36^. Fortunately Raman and PL spectroscopy can confirm layer thickness with confidence^37^. AFM in this case serves as a higher resolution microscope to resolve finer details otherwise invisble under an optical microscope, such as the presence of a crack. The Raman spectrum plotted in Fig. 1b, obtained with 633 nm laser excitation shows the ${\text{E}}_{2\text{g}}^{1}$ and ${\text{A}}_{1\text{g}}$ peaks with a mode separation of $61 {\text{ cm}}^{-1}\pm 2{\text{ cm}}^{-1}$, which confirms that this is a sample of monolayer WS_2_^38^.

The room temperature PL spectrum of single layer WS_2_, plotted in Fig. 1c, is dominated by the coulomb-bound free exciton transition (X0) at approximately 2.01 eV, these excitons have a high thermal stability due to their large binding energy^39^. Stable three-particle states, in the presence of excess carrier population of electrons or holes, can also form and are known as charged excitons (CX) or trions, whose binding energy corresponds to a energetic-redshift relative to the free exciton. The n-type^26,40^ nature of as-exfoliated WS_2_ implies that CX transitions are negatively charged due to excess electrons. The excess electron density can be controlled by the application of a gate voltage, whereupon an increase in excess electron density leads to a strengthening of the CX state and weakening of the X0 state^41^. In our study, the excess electron density is expected to orginate from the naturally occuring atomic defects and impurities present in the lattice, which can donate electrons via, for instance, photo-ionisation^34,42^. Defects can also serve as trapping potentials and can lead to localised excitons (LX). This simplified picture of the optically allowed transitions in monolayer WS_2_ considers just the transitions within one of the K and K’ pseudo-spin valleys^43^. At room temperature the PL contributions from excitons, charged excitons, and localised excitons overlap strongly but can be separated through careful analysis of the PL spectrum. The supplementary materials section includes a detailed discussion on the fitting procedures applied in this paper. The relative contributions of these excitonic species to the overal PL emission (i.e. the spectral weightings) are indicative of optical quality measures such as the PL yield, for instance weaker PL intensity is associated with large spectral weightings of the charged exciton^38,44^.

Here the spatial homogeneity of the exciton spectral weightings e.g. for the charged exciton, ${\text{S}}_{\text{CX}}= {\text{I}}_{\text{CX}}/({\text{I}}_{\text{X}0}+ {\text{I}}_{\text{CX}} + {\text{I}}_{\text{LX}})$ are being considered. Application of the mass-action law under thermal equilibrium assumptions for a laser-illuminated monolayer MoS_2_ leads to a simple relationship between ${S}_{CX}$ and the density of excess charge carriers^41^. An alternative approach exists that relates the charge density to the ratio of intensities between the X0 and the CX transitions rather than the charged exciton spectral weighting^45^. The methods presented here to quantify spatial homogeneity are applicable to either approach.

### Point-spectroscopy and population modelling

**Figure 2:**
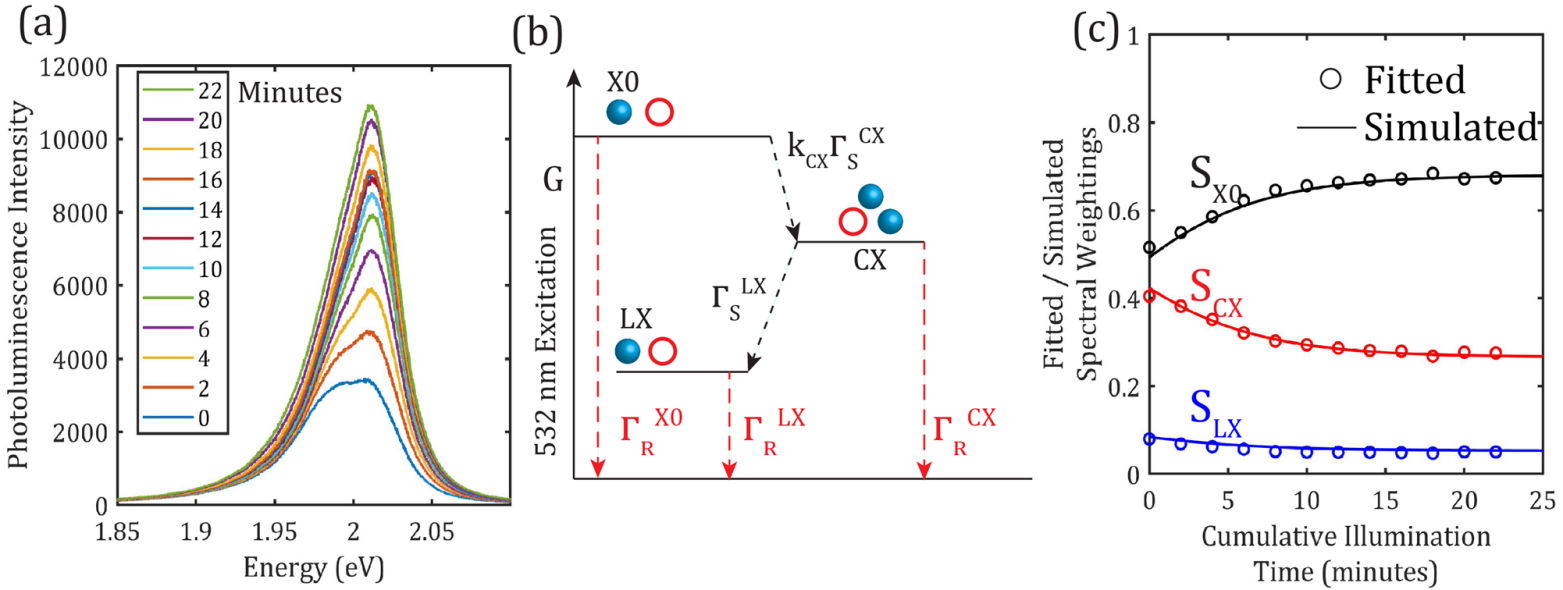
(**a**) PL spectrum series measured over 22 min laser illumination showing the evolution of the PL spectrum over time ${I}_{L}=6 kW{cm}^{-2}$, ${t}_{i}=1 s$. (**b**) Diagram illustrating model used to describe dynamics of excited state populations, corresponding with Eqs. (1–5) (**c**) Fitted spectra weightings of exciton (X0), charged exciton (CX), and localised exciton (LX) emission (plotted as circles) compared with simulated data (plotted with lines) obtained using the proposed model. Error bars are estimated to be smaller than the data points, more on this can be found in the supplementary materials.

Whilst PL spectroscopy offers a sensitive tool for characterisation of monolayer WS_2_, it is known that the associated-illumination can also result in changes in the observed PL emission. This is exemplified in Fig. 2a, which shows a series of PL spectra measured at regular intervals during illumination of a WS_2_ monolayer flake over 22 min with a $532 \text{nm}$ wavelength laser ($6 {\text{kWcm}}^{-2}$). This series of spectra exhibits a monotonic increase in the intensity of the PL emission, primarily due to an enhancement of emission from the excitonic emission centred around $2.01 \text{eV}$. By fitting each of these spectra as described above, it is possible to extract spectral weightings for the different excitonic contributions ${\text{S}}_{\text{X}0}$, ${\text{S}}_{\text{CX}}$, and ${\text{S}}_{\text{LX}}$. These are plotted against laser illumination time in Fig. 2c and show that with increased illumination-time, the excitonic emission strengthens, whereas the charged exciton and localised exciton contributions decrease. For all three species, the rate of change is initially high and decreases over time towards apparently asymptotic values for the spectral weightings.

In order to interpret these changes in the PL emission we have adapted a population model describing the system in terms of the ground state, neutral exciton, charged exciton, and localised exciton. A coupled rate equation model in the first order describes the kinetics of the population*s *${P}_{X0}$*,* ${P}_{CX}$ *and* ${P}_{LX}$, corresponding to the exciton, charged exciton, and localised exciton states. This model is illustrated in Fig. 2b and described by Eqs. (1–5), where Eq. (5) calculates the spectral weightings ${S}_{m}$, with subscripts $m:[X0,CX,LX]$, based on the steady state solutions, these equations were adpated from a different study^46^ on WS_2_ dressed MoS_2_. The proposed equations, and corresponding steady state ($\dot{P}=0$) solutions are listed below. Scattering from exciton to charged exciton ${\Gamma }_{S}^{CX}$ is not radiative and neither is ${\Gamma }_{S}^{LX}$, large scattering rates will reduce the pathways for radiative recombination and thus photoluminescence yield.

1 $$ \mathop {{\text{P}}_{{{\text{X}0}}} }\limits^{.}  = {\text{G}} - {\text{P}}_{{{\text{X}}0}} \left( {\Gamma _{{\text{R}}}^{{{\text{X}}0}}  + \Gamma _{{\text{S}}}^{{{\text{CX}}}} {\text{k}}_{{{\text{CX}}}} } \right)\qquad{\text{P}}_{{{\text{X}}0}}  = \frac{{\text{G}}}{{{\text{k}}_{{{\text{CX}}}} \Gamma _{{\text{S}}}^{{{\text{CX}}}}  + \Gamma _{{\text{R}}}^{{{\text{X}}0}} }} $$

2 $$ \mathop {{\text{P}}_{{{\text{CX}}}} }\limits^{.}  = {\text{P}}_{{{\text{X}}0}} \Gamma _{{\text{S}}}^{{{\text{CX}}}} {\text{k}}_{{{\text{CX}}}}  - {\text{P}}_{{{\text{CX}}}} \left( {\Gamma _{{\text{R}}}^{{{\text{CX}}}}  + \Gamma _{{\text{S}}}^{{{\text{LX}}}} } \right)\;\qquad{\text{P}}_{{{\text{CX}}}}  = \frac{{{\text{P}}_{{{\text{X}}0}} \Gamma _{{\text{S}}}^{{{\text{CX}}}} {\text{k}}_{{{\text{CX}}}} }}{{\Gamma _{{\text{S}}}^{{{\text{LX}}}}  + \Gamma _{{\text{R}}}^{{{\text{CX}}}} }}, $$

3 $$ \mathop {P_{{{\text{LX}}}} }\limits^{.}  = {\text{P}}_{{{\text{CX}}}} \Gamma _{{\text{S}}}^{{{\text{LX}}}}  - {\text{P}}_{{{\text{LX}}}} \Gamma _{{\text{R}}}^{{{\text{LX}}}} \qquad {\text{P}}_{{{\text{LX}}}}  = \frac{{{\text{P}}_{{{\text{CX}}}} \Gamma _{{\text{S}}}^{{{\text{LX}}}} }}{{\Gamma _{{\text{R}}}^{{{\text{CX}}}} }},$$

4 $${\text{k}}_{\text{CX}}\left(\text{t}\right)={\text{k}}_{\text{CX}}^{0}\text{exp}(-\text{G}\times \text{t})+\upbeta $$

5 $$ {\text{S}}_{{\text{m}}}  = \frac{{\Gamma _{{\text{R}}}^{{\text{m}}} {\text{P}}_{{\text{m}}} }}{{\Gamma _{{\text{R}}}^{{{\text{CX}}}} {\text{P}}_{{{\text{CX}}}}  + \Gamma _{{\text{R}}}^{{{\text{X}}0}} {\text{P}}_{{{\text{X}}0}}  + \Gamma _{{\text{R}}}^{{{\text{LX}}}} {\text{P}}_{{{\text{LX}}}} }} $$

$G$ is the exciton generation rate associated with the optical excitation of the sample and is constant if the laser intensity does not change during the measurement. ${\Gamma }_{R}^{X0}$, ${\Gamma }_{R}^{CX}$, and ${\Gamma }_{R}^{LX}$ are radiative recombination rates for the corresponding excited states. ${\Gamma }_{S}^{CX}$ and ${\Gamma }_{S}^{LX}$ are scattering rates (see Fig. 2b) describing the formation of charged excitons and localised excitons, respectively. The rate at which excitons (X0) capture electrons from donor defects to form charged excitons (CX) depends on the availability of such defects, parameterised by ${k}_{CX}$, written as a dimensionless pre-factor for ${\Gamma }_{S}^{CX}$. It is hypothesized that ${k}_{CX}$ decays exponentially with time, on the order of seconds to minutes and assumed to be a function of the exciton generation rate $G$, with parameters ${k}_{CX}^{0}$ and $\beta$. ${k}_{CX}$ is used in similar ways in other experimental models of controlled photo- and chemical-doping of monolayer TMDs^46,47^, except that, as we show later, it potentially carries spatial dependence as well as time- (or processing step) dependence. The reduction in ${k}_{CX}$ then corresponds to a reduction in the nonradiative scattering rate from excitons to charged excitons, which naturally enhances the emission yield from the exciton state. The localised excitons (LX) can form and decay radiatively in TMDs, due to either collision between photo-generated excitons and sub-bandgap defects^48^, dissociation of CX states via defects^46^ or excited directly under near-resonant conditions^49^. Elucidating a specific mechanism for the formation of the LX state is unrealistic at room temperature, therefore for simplicity it was assumed that localised excitons form via the charged state, based on a recent report^46^. Although somewhat speculative, it is reasonable and Fig. 2c suggests the localised exciton plays a minor role compared to the contributions from CX and X0 transitions.

Equations (1) to (3) enable us to solve for the populations of the various exciton species under steady state conditions, but to incorporate the experimentally observed dependence of the spectral weighting with laser illumination time, we propose the further introduction of a time-dependence of ${k}_{CX}(t)$, which is given in Eq. (4). This time-dependence describes the observed changes in PL spectral weighting with post-processing sample treatment, which in our study is photo-induced via controlled laser illumination. Here we have described this process as a generalised exponential decay of the defect availability. Other reports of controlled environmental doping of TMDs consider an inverse relationship with for instance the concentration of dopants, in step-wise application of chemical treatments^44,46,47,50–52^ or laser-annealing^34^ that modifies the density of excess charge-carriers and hence the spectral weightings of excitons and charged excitons**.** The exponential dependence is chosen here empirically in order to achieve a good fitting of the experimental data, as shown in Fig. 2c, a further validation of this exponential decay is provided later in the manuscript, with consideration of spatial-homogeneity.

The radiative constants used in the model ${\Gamma }_{R}^{X0}=0.002 {\text{ps}}^{-1}$,${\Gamma }_{R}^{CX}=0.01 {\text{ps}}^{-1}$, ${\Gamma }_{R}^{LX}=0.01 {\text{ps}}^{-1}$, are slightly smaller than those obtained from transient absorption studies of monolayer MoS_2_ on silicon substrates^53^, probably due to the lower dielectric constant of the PDMS substrate compared to silicon dioxde^54^. It was assumed that these values are invariant under laser-induced changes. The intial value of ${S}_{CX}\sim 0.4$, shown in Fig. 2c, is associated with the defect availability ${k}_{CX}\approx 1.9$. As the illumination time increases ${k}_{CX}$ reduces to a final value of approximately 0.95, which effectively reduces ${\Gamma }_{\text{S}}^{\text{CX}}$ and hence the spectral weighting of the charged exciton ${S}_{CX}$. During this process ${S}_{X0}$ is enhanced and the overall PL intensity increased (by ~ 230% after 22 min of illumination). Dimensionless parameters ${k}_{CX}^{0}\approx 0.8$ and $\beta \approx 0.7$ were found to give the best fit along with scattering parameters ${\Gamma }_{S}^{CX}$*/*${\Gamma }_{S}^{LX}\approx 0.5$, with ${\Gamma }_{S}^{CX}$=0.001 ${\text{ps}}^{-1}$.

### Spatial homogeneity dynamics and enhancement

**Figure 3:**
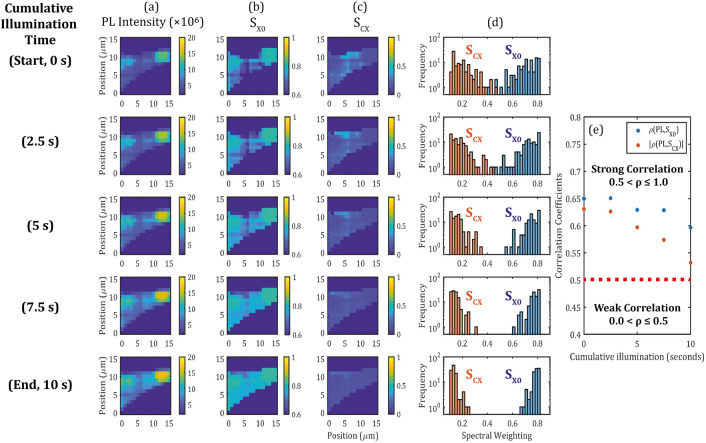
Illumination-time dependence for a small monolayer region studied using time-stepped PL maps. (**a**) Fitted PL intensity, (**b**) ${S}_{X0}$ and (**c**) ${S}_{CX}$ maps extracted at different laser illumination times. From top to bottom: $t=0 s$ (before laser illumination) and after repeated laser illumination time steps $t=2.5 s$, $5 s$, $7.5 s$ and $10 s$. Histograms of ${S}_{CX}$ and ${S}_{X0}$ values from each map are plotted in column (**d**). Correlation statistics between the PL intensity and each of the spectral weighting maps ${S}_{CX}$ and ${S}_{X0}$ are extracted and plotted against the laser illumination time in (**e**), with the absolute value taken for $\rho (PL,{S}_{CX})$.

It can be shown that the parameter ${k}_{CX}$ carries a spatial dependence in addition to the macroscopic time-dependence, by applying spatial statistics on PL maps that accounts for the spatial inhomogeneity. In this measurement, a separate monolayer flake is chosen and the laser power is increased to $78\text{ kW}{\text{cm}}^{-2}$, from $6\text{ kW}{\text{cm}}^{-2}$ in the previous measurement, which is expected to increase the rate of change of the spectral weightings^44,51^. Rapid spectral tuning is crucial for scalability as this will reduce the overall treatment time of large-area flakes. For each spatial position, continuous illumination ($78\text{ kW}{\text{cm}}^{-2}, {\text{t}}_{\text{i}}=2.5\text{ s})$ and PL measurement ($78\text{ kW}{\text{cm}}^{-2}, {\text{t}}_{\text{i}}=100\text{ ms})$ were alternated to obtain five spectral maps, corresponding to $\text{t}=0, 2.5, 5, 7.5, 10\text{ s}$ respectively, shown in Fig. 3 column (a) where $\text{t}=0\text{ s}$ and $\text{t}=10\text{ s}$ are the initial and final PL measurements. The scanning resolution was set at 1 µm in both spatial directions. Corresponding ${\text{S}}_{\text{X}0}$ and ${\text{S}}_{\text{CX}}$ maps were then extracted using fitting and are plotted in Fig. 3 column (b) and column (c) respectively. The histograms for these two parameters are also plotted corresponding to the time-step in Fig. 3 column (d). The localised exciton plays a minor role in shaping the PL spectrum, thus its statistics are not shown here. Spatial-statistics of these maps are provided in terms of correlation coefficients of the multivariate (PL intensity, ${\text{S}}_{\text{CX}}$) and (PL intensity, ${\text{S}}_{\text{X}0}$), as $\rho (PL,{S}_{CX})$ and $\rho (PL,{S}_{X0})$ respectively, plotted in Fig. 3e are off-diagonal elements of the $2\times 2$ correlation matrices between the PL intensity and each of the spectral weightings, respectively. Since $\rho (PL,{S}_{CX})$ is an anti-correlation set with negative coefficients, its absolute value is plotted here to more easily compare with the rate of change in $\rho (PL,{S}_{X0})$ with cumulative laser-illumination time.

At the start of the treatment ($t=0 s$), both the PL intensity and spectral weighting maps show a high degree of inhomogeneity, i.e. large variance. This is also reflected in the spectral weighting histogram in Fig. 3d, in which ${S}_{CX}$ and ${S}_{X0}$ values are spread over large and nearly overlapping ranges ($\overline{{S}_{CX}}(t=0\text{ s})=0.20\pm 0.08$ and $\overline{{S}_{X0}}(t=0\text{ s})=0.71\pm 0.09$, with uncertainties expressed as standard deviation with coverage factor. With increasing laser illumination time, on average not only ${S}_{CX}$ decreases and ${S}_{X0}$ increases but the distribution of values (i.e. inhomogeneity) also reduces significantly. By the end of the 10 s laser illumination, ${S}_{CX}$ and ${S}_{X0}$ reach mean values of $\overline{{S}_{CX}}(t=10 \text{s})=0.14\pm 0.03$ and $\overline{{S}_{X0}}(t=10 \text{s})=0.79\pm 0.03$, which represents a threefold reduction in standard deviation, when compared to before the laser illumination. Assuming that the decay rate of the availability parameter ${k}_{CX}$ is invariant under the same laser power, then only a reduction of the mean spectral weighting is expected, not of its variance. The reduction in variance may be accounted for by a differential sensitivity to laser illumination across the sample, which is considered next.

The initial inhomogeneity (statistical variance) in ${\text{S}}_{\text{X}0}$ (${\text{S}}_{\text{CX}}$) is strongly correlated (anti-correlated) with the initial inhomogeneity in the PL intensity with ${\uprho }_{\text{PL},\text{Sx}0}=0.65$. With increasing illumination time, however, the strength of these correlations decreases, with ${|\uprho }_{\text{PL},\text{Scx}}|$ changing faster than $|{\uprho }_{\text{PL},\text{Sx}0}|$. This indicates that the laser illumination reduces the contribution of charge-based inhomogeneity to the observable PL inhomogeneity. Figure 3e suggests that at longer laser-illumination times, the ${|\uprho }_{\text{PL},\text{Scx}}|$ correlation would become weaker (< 0.5) and tend towards the zero-correlation limit. In this limit, charge-inhomogeneities would be effectively decoupled from the PL inhomogeneities and any remaining inhomogeneity could be indicative of alternative factors such as macroscopic defects. Apart from illumination-time, the laser power is also a crucial factor. The reduction in spectral weighting is evidently faster at higher laser power. During the low power measurement presented in Fig. 2 ($6\text{ kW}{\text{cm}}^{-2}$), ${S}_{CX}$ decreased by 30% in 22 min, compared to the measurement presented in Fig. 3 ($78\text{ kW}{\text{cm}}^{-2}$), where ${S}_{CX}$ decreased by 30% in approximately 10 s. Keeping the same illuminating time but using even higher laser powers will eventually lead to damage^55^ above a certain threshold, which was observed at around $100\text{ kW}{\text{cm}}^{-2}$ for the samples studied here. Longer illumination time at constant laser power is anticipated to reduce the mean and the variance of the spectral weighting further towards an asymptotic minimum.

**Figure 4:**
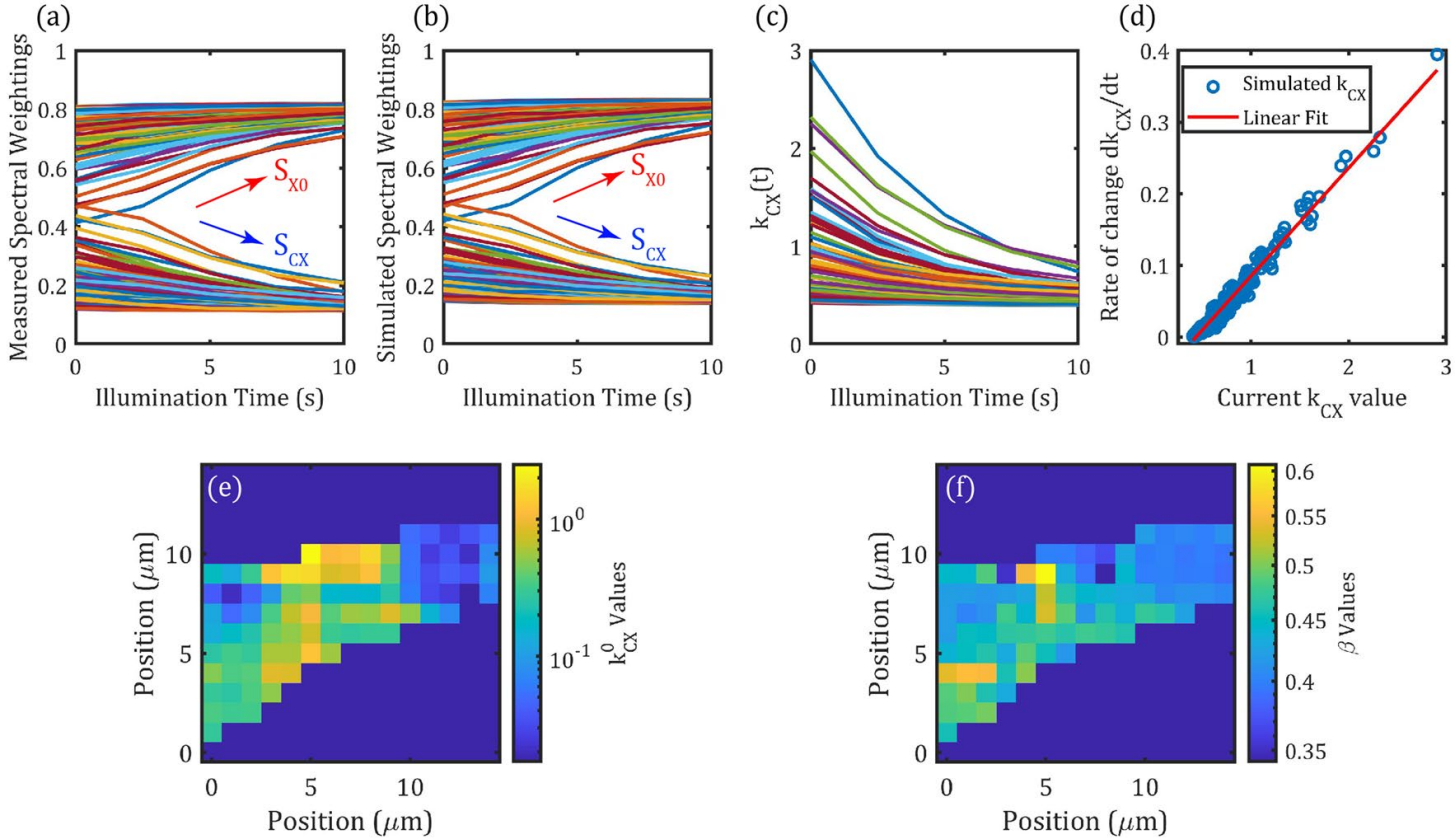
Spatial–temporal analysis of spectral weighting changes. (**a**) Spectral weighting obtained from Voigt fitting for all pixels, plotted against the corresponding illumination time. (**b**) Spectral weighting obtained from the steady state model after optimisation step to obtain ${k}_{CX}(x,y,t)$. (**c**) ${k}_{CX}$ plotted as a function of illumination time, for all spatial locations. (**d**) The rate of change of ${k}_{CX}$(t) plotted against its instantaneous value at all time steps and all spatial locations. Linear fit shows that ${k}_{CX}(t)$ can be described by exponential decay, fitting the exponential form of ${k}_{CX}$ from Eq. (4) leads to the extraction of two parameters ${k}_{CX}^{0}$ and $\beta$ for each pixel, visualised as maps in (**e**) and (**f**) respectively with logarithmic color scales for ${k}_{CX}^{0}$.

The spatial measurements presented in Fig. 3 suggest that the parameter ${k}_{CX}$ should be a function of the spatial positions as well as time. While it was formulated based on equations with respect to time, it can be extended to the spatial domain by fitting the steady-state PL model for each position on the monolayer. Figure 4a plots together the step-wise change to the fitted neutral exciton and charged exciton spectral weightings ${S}_{X0}$, ${S}_{CX}$ for each pixel in the mapped positions shown in Fig. 3. Qualitatively, changes in spectral weighting agree with the single-point PL measurement, with ${S}_{CX}$ decreasing and ${S}_{X0}$ increasing with laser-illumination time. For each sampled spatial position, a time-vector of ${k}_{CX}$ values was calculated by fitting the predicted spectral weightings from the steady-state model to the measured spectral weightings in time. This essentially produces a ${k}_{CX}$ vector for each spatial position and allows the steady-state model to simulate the spectral weighting dynamics extended to all spatial positions. Figure 4b shows that this simulated spatial-dynamics reproduces the measurement (Fig. 4a) well, providing some validation for the spatial-time fittings. The time-dependence of ${k}_{CX}$ for all pixels is shown in Fig. 4c. We find a linear relationship when plotting its derivative against its instantaneous value, shown in Fig. 4d for each illumination step, justifying the exponential form of ${k}_{CX}\left(t\right)={k}_{\mathit{CX}}^{0}\mathit{exp}(-G*t)+\beta$. With $\text{G}=2.6$, parameter maps corresponding to ${k}_{CX}^{0}$ and $\beta$ at each spatial location can be found by fitting this exponential decay. These maps are displayed in Fig. 4e,f, respectively.

Whilst ${k}_{CX}^{0}$ and $\beta$ do not have simple physical interpretations, they can be understood as the contributions to the overall defect availability that respond to laser illumination (${k}_{CX}^{0}$) and those that do not ($\beta$). Regions with higher values of ${k}_{CX}^{0}$ undergo larger changes in spectral weighting during the same illumination-time compared with regions with lower ${k}_{CX}^{0}$ values. It is assumed that ${k}_{CX}^{0}$ indicates the initial availability of defects that can be photo-ionized and it is clear from Fig. 4e that a considerable spatial variation of this parameter exists. This inhomogeneity in ${k}_{CX}^{0}$ is strongly correlated (anti-correlated) with ${S}_{CX}$(${S}_{X0}$). There is also an inhomogeneity in values of $\beta$ across the flake, though its correlation with ${S}_{CX}$ is much lower. As such, $\beta$ could be indicative of defects that do not respond to changes induced by laser-illumination on the monolayer WS_2_ in ambient-conditions, such as as macroscopic defects in the flake.

**Figure 5:**
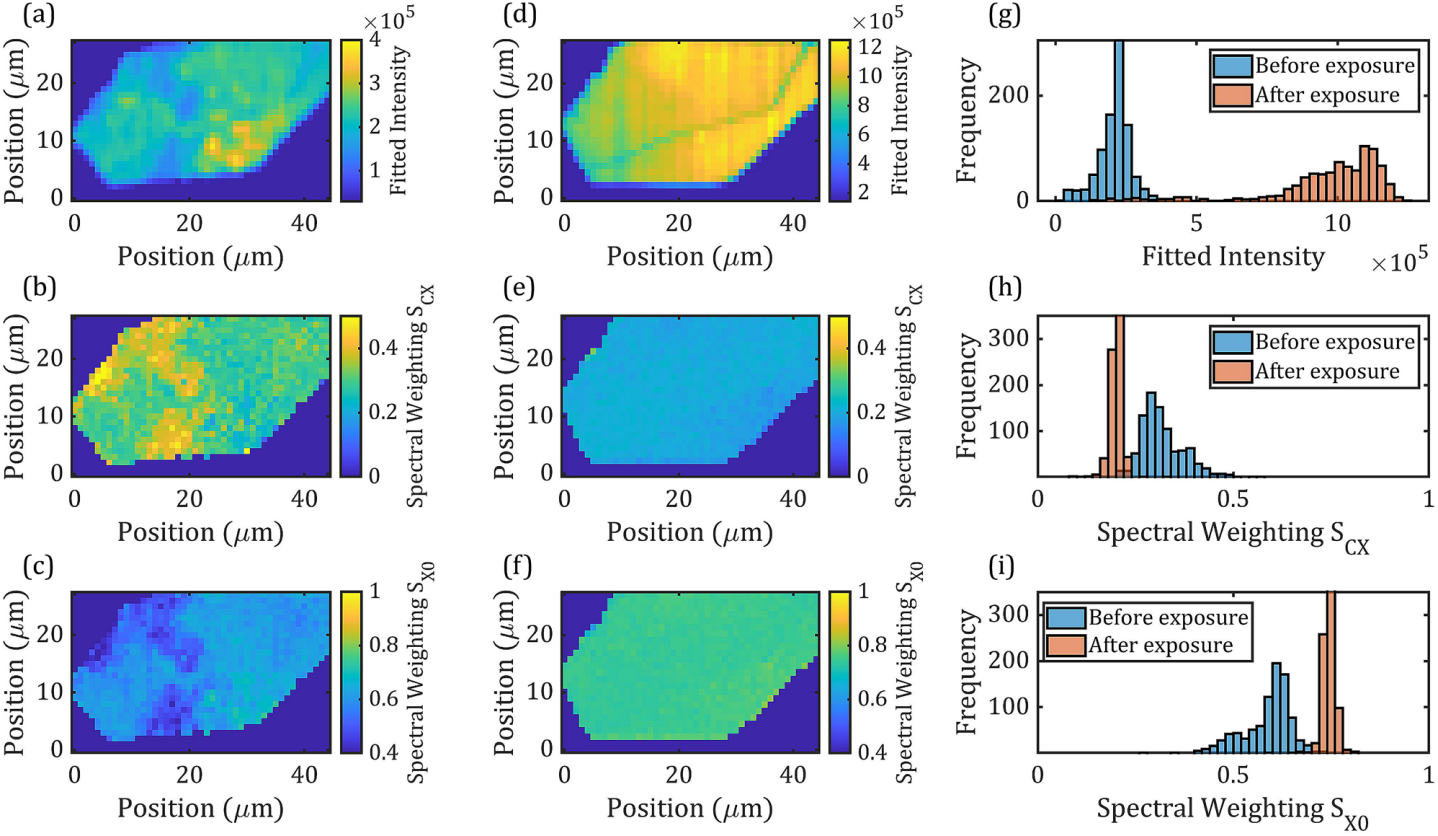
Maps and statistics from large area WS_2_ flake illuminated with high power (${I}_{L}=78 kW{cm}^{-2}$, 10 s) laser in raster-scan. PL maps are acquired before (**a**) and after (**d**) the laser illumination, ${S}_{CX}$ and ${S}_{X0}$ maps were extracted from modelling the PL maps both before (**b**,**c**) and after (**e**,**f**) the laser illumination. Histograms of the PL peak intensity (**g**), charged exciton (**h**) and exciton (**i**) spectral weightings before and after the laser illumination are then plotted.

To further demonstrate the reproducibility of the effect of laser treatment and evaluate the possibility of a single illumination step process, we applied the laser treatment to the larger WS_2_ monolayer flake shown in Fig. 1. A single illumination step of 10 s was applied with the laser intensity kept at ${\text{I}}_{\text{L}}=78 {\text{kWcm}}^{-2}$. PL maps were recorded with low laser intensity ${\text{I}}_{\text{L}}=2.5 {\text{kWcm}}^{-2} ({\text{t}}_{\text{i}}=1\text{s}$ illumination) before- and after-laser treatment to avoid further changes to the PL. These maps are shown in Fig. 5 along with extracted spectral weighting maps. Only histogram statistics are shown here for simplicity. There is an enhancement of the overall PL intensity (~ 300% in this case) and a significant reduction (enhancement) in the CX (X0) spectral weighting Fig. 5e,f. These results are summarised and compared with the multi-step illumination experiment in Table 1. Recent reports^38,56^ on the PL of monolayer WS_2_ showed pronounced exciton emission at the edges the exfoliated flakes. Here, the upper-left corner of the flake in Fig. 5b, which appears wrinkled in the microscopy measurement (Fig. 1), corresponds with pronounced charged exciton emission, which we assign to mechanical strain^57^. Since Fig. 5e,f shows highly homogeneous exciton and charged exciton spectral weighting distributions after laser-illumination so we conclude that flake edges do not undergo qualitatively different spectral changes under continuous laser illumination compared to the interior parts of the flake.

**Table 1:** Comparison of the results from the two homogenisation experiments.

Experiment	Illumination time (s)	Approximate flake area	$\overline{{S}_{CX}}$ relative change	$\overline{{S}_{X0}}$ relative change
1	$2.5 s\times 4$	$120 {\mu m}^{2}$	$-30\text{ \%}$	$+11\text{ \%}$
2	$10 s\times 1$	$1050 {\mu m}^{2}$	$-38\text{ \%}$	$+25\text{ \%}$

The relative changes in ${S}_{CX}$ and ${S}_{X0}$ are computed from the averages of all pixels taken from the before- and after-illumination PL maps for experiment 2 and $t=0s$ and $t=10s$ PL maps for experiment 1.

The absolute changes in ${S}_{CX}$ and ${S}_{X0}$ are on average twice as high in the treatment of the larger flake (Flake 2), compared to the smaller flake (Flake 1), which correlates with the higher initial ${S}_{CX}$ values in Flake 2. Indeed, as we observed in Fig. 4, a strong positive correlation ($\rho =0.992$) can be found between the initial ($t=0$) ${S}_{CX}$ weighting values and the corresponding rate of change of ${S}_{CX}$ over the 10 s illumination time. Positions on the monolayer flake with high spectral proportion of charged excitons are more likely to undergo a significant change to the PL over the same period of laser illumination time. This differential “sensitivity” across the flake accounts for the reduction in the variance, i.e. the inhomogeneity of spectral weightings. However, if the laser illumination would remove all defects that impact the PL emission in WS_2_ flakes, then we would expect the homogeneity of ${S}_{CX}$ to directly lead to homogenisation of the PL intensity. However, Figs. 5g–i shows that the histograms of the PL intensity remains almost unchanged despite the increased overall PL intensity and a fivefold reduction in the variance of ${S}_{CX}$. That fact that the homogeneity can be improved is evidence that supports the spatial-dependent model presented earlier, pointing to changes in the monolayer rather than spatially invariant effects, e.g. thermal annealing^58^ on the substrate.

As discussed in Fig. 3e, for longer laser illuminations, we expect the decrease in correlation between spectral weighting and PL intensity homogeneity to highlight additional defects that may have been difficult to identify before the treatment. Apart from charge-defects, which are intrinsic to the crystal lattice, other extrinsic defects may also contribute to the PL inhomogeneity, including topological defects (cracks, folds, ripples, bubbles) that reduce quantum yield, and other charge-transfer mechanisms between the WS_2_ and its dielectric environment. From Fig. 5a–d the appearance of a thin PL intensity discontinuity corresponding to the crack observed in Fig. 1a suggests that the laser-illumination effectively decouples spectral inhomogeneity based on charge-phenomena, from other defects, which may be topological in this case, potentially improving their visibility in optical images. A repeat of this measurement was performed after the flake had been left in ambient conditions for 5 months. This data is presented in SM-Fig. S7, which shows that the variance has recovered towards its initially broad distribution, whereas the mean of the spectral weightings has stayed approximately unchanged. This partial-reversibility indicates a combination of reversible removal of surface adsorbates^44^, but also an irreversible (at least on this timescale) change to the concentration of excess carriers, suggesting chemical passivation of defects.

The laser-illumination method to enhance spectral homogeneity presented here impacts the excess charge carrier concentration in the monolayer WS_2_ on PDMS, this is also observed from point-spectroscopic studies using SiO_2_/Si^33^ and h-BN/SiO_2_/Si^34^ substrates. While qualitatively similar observations on different substrates can be expected, the extent of enhancement, e.g. the rate of change of spectral weighting at similar laser power and illumination time, may be different, depending on how much of the spatial inhomogeneity is from charge-phenomena in the first place.

## Conclusion

A simple laser treatment in ambient conditions was shown to minimise the generation of charged excitons and enhance the population of neutral excitons leading to increased PL yield and improved homogeneity, which is interpreted as resulting from spatially and time- (process-) dependent charged exciton generation rates across the TMD. The demonstrated reduction in spectral inhomogeneity by up to five-times, has applications in areas such as photonics, where cavity interaction but may also reduce the Fermi-level pinning effect, cause by electronic inhomogeneity. Moreover, as this technique does not rely on encapsulation or suspension, the atomically sensitive surface remains intact and would be readily applicable for molecular sensing applications, including on flexible substrates.

A statistical method for quantifying the spatial inhomogeneity of WS_2_ material quality pertaining to optical and electronic transport phenomena was proposed. Measurements of the spectral homogeneity is a reliable way to characterise and quantify changes to the material’s optoelectronic properties in-between different processes, such as transfers, chemical doping, stacking etc. Improving the spectral homogeneity can also isolate inherent electronic defects from other factors contributing to spatial-inhomogeneity related performance-limitation in TMD-devices, including substrates^17,59^ and TMD-metal interfaces^16,18,19^. The measurement and modelling of spatial dynamics presented here is the first of its kind, as far as the authors are aware, and should be beneficial and applicable to future studies on other processes that changes the spatial homogeneity.

## Methods

Mechanical Exfoliation. For this study, a monolayer WS_2_ sample was prepared from a tungstenite crystal (HQ Graphene Ltd.) using mechanical exfoliation. Low-residue tape (Nitto BT-150P-LC) containing bulk crystals was laminated onto low-tack polydimethylsiloxane (PDMS, GelPak PF-70-X4) films, then peeled off, leaving flakes of various thickness on the stamp.

Atomic Force Microscopy. WS_2_ flake topography was measured on an AIST-NT Combiscope 1000. Sharp silicon nitride probes (Bruker, SNL, Lever C) with low nominal spring constant of $0.24 \text{N}/\text{m}$ were used to record high-resolution surface topography after PL characterisation. Contact mode was applied, a low force set-point was used to avoid modification of the sample surface.

Photoluminescence and Raman Spectroscopy. WS_2_ flakes were analysed using a Horiba LabRAM Evolution spectrometer with a $532 \text{nm}$ excitation laser. The excitation-intensity was controlled using neutral-density filters mounted to a rotator and using the diode current controls on the laser driver. The laser intensity delivered to the sample, through a 100 × 0.9 numerical aperture objective lens, was measured using a calibrated Thorlabs S120VC photodiode. The beam profile was assumed to be Gaussian with a diffraction limited minimum focal spot with diameter $D = 1.22\lambda /NA \approx 721 \text{nm}$, where $\lambda$ is taken as $532 \text{nm}$. 2D confocal µ-PL maps were acquired by scanning the sample position with respect to a fixed laser focus, using $x-y$ linear positioners. Raman spectroscopy was carried out using the same objective lens as photoluminescence spectroscopy except that $633 \text{nm}$ laser excitation was used to excite the Raman modes of the monolayer WS_2_. Luminescence background was removed prior to spectral analysis.

Time-series PL maps. Using a Horiba LabRAM instrument, a time-$XY$ map was setup, with $XY$ being the spatial co-ordinates of the map and time being the laser-illumination time at each $XY$ position. The homogeneity enchancement measurements were carried out by illuminating a point with spatial coordinates (${x}_{0},{y}_{0}$) continuously over some illumination-time. Then the same illumination cycle was applied to the next points (${x}_{1...n},{y}_{1...m}$) until an array of positions ($m \times n$) had all received the same illumination-time. In the measurement presented in Fig. 3, the illumination-time was $2.5 \text{s}$ and repeated 4 times to give $10 \text{s}$ of cumulative illumination-time. In the measurement presented in Fig. 5, the illumination-time was $10 \text{s}$.

## Supplementary Information

Supplementary Information.

## Data Availability

The data that support the findings of this study are available from the corresponding author upon reasonable request.

## Code availability

The codes that support the findings of this study, detailed in this paper are available from the corresponding author upon reasonable agreement.
