# Author Correction: Enhancing and quantifying spatial homogeneity in monolayer WS_2_

**DOI:** 10.1038/s41598-021-97594-9

**Published:** 2021-09-06

**Authors:** Yameng Cao, Sebastian Wood, Filipe Richheimer, J. Blakesley, Robert J. Young, Fernando A. Castro

**Affiliations:** 1 National Physical Laboratory, Hampton Road, Teddington, TW11 0LW UK; 2 Department of Physics, Lancaster University, Lancaster, LA1 4YB UK; 3 Advanced Technology Institute, University of Surrey, Guildford, Surrey, GU2 7XH UK

Correction to: *Scientific Reports* https://doi.org/10.1038/s41598-021-94263-9, published online 21 July 2021

The original version of this Article contained an error in Figure 5 where panel (e) was incorrectly captured. The original Figure 5 and accompanying legend appear below.

**Figure 5:**
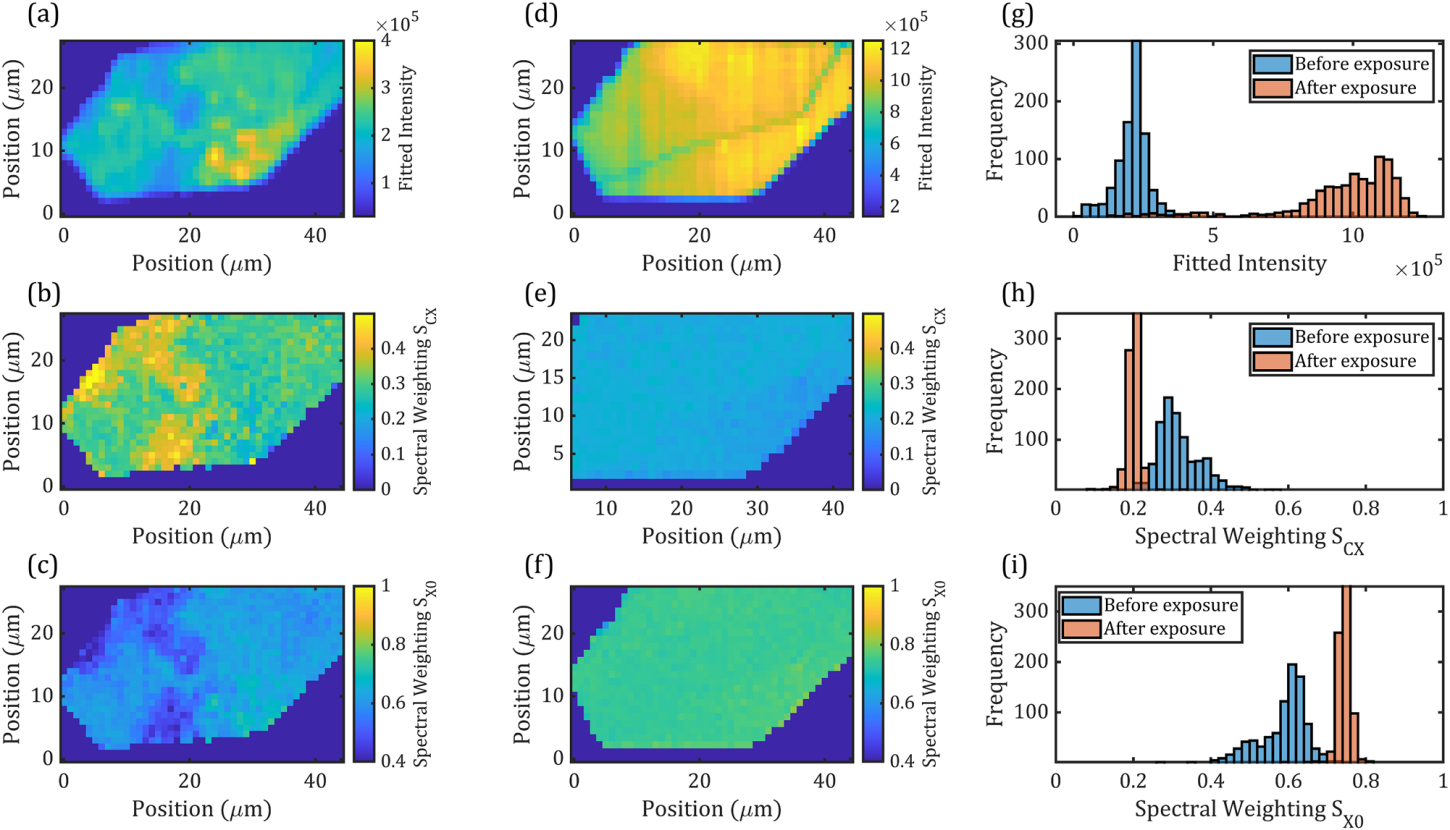
Maps and statistics from large area WS_2_ flake illuminated with high power (*I*_*L*_ = 78*kWcm*^−2^, 10 s) laser in raster-scan. PL maps are acquired before (**a**) and after (**d**) the laser illumination, *S*_*CX*_ and *S*_*X*0_ maps were extracted from modelling the PL maps both before (**b**,**c**) and after (**e**,**f**) the laser illumination. Histograms of the PL peak intensity (**g**), charged exciton (**h**) and exciton (**i**) spectral weightings before and after the laser illumination are then plotted.

The original Article has been corrected.

