# Supplementary material for: Enhancing and quantifying spatial homogeneity in monolayer WS2
Source: Sci Rep. 2021 Jul 21;11:14831. doi: 10.1038/s41598-021-94263-9 (PMC8295334; doi:10.1038/s41598-021-94263-9)
Supplement: Supplementary file 1 — Supplementary Information. [file 41598_2021_94263_MOESM1_ESM.pdf]

Enhancing and quantifying spatial homogeneity in monolayer WS_2

Supplementary Materials

Yameng Cao[1,*], Sebastian Wood[1], Filipe Richheimer[1], J. Blakesley[1], Robert J. Young[2], Fernando A. Castro[1,3]

[1] National Physical Laboratory, Hampton Road, Teddington, TW11, 0LW, United Kingdom
[2] Department of Physics, Lancaster University, Lancaster, LA1 4YB, United Kingdom
[3] Advanced Technology Institute, University of Surrey, Guildford, Surrey, GU2 7XH

E-mail[*]: yameng.cao@npl.co.uk

1. Three-Voigt Model

We used the three-Voigt Model to describe the spectral shape of single layer WS_2 at ambient conditions. We justify this here.

The spectral line shape of bound excitons results from optical transitions that decay exponentially in time. In the frequency space, this is the Lorentzian function in the case of a truly isolated emitter. For well-isolated systems often, the Lorentzian approximation is good enough for the intrinsic linewidth, but thermal effects bring with it a Gaussian component. The Voigt profile. This is given by,

$$\phi(x,y) = \frac{1}{\alpha_G}\sqrt{\frac{\ln 2}{\pi}}K(x,y),$$

$$K(x,y) = \frac{x}{\pi}\int_{-\infty}^{+\infty}\frac{\exp(-t^2)}{y^2+(x-t)^2}dt$$

Here, x and y are given by,

$$x = \frac{(\upsilon-\upsilon_0)\sqrt{\ln 2}}{\alpha_G}, y = \frac{\alpha_L\sqrt{\ln 2}}{\alpha_G}$$

This contains the Gaussian and Lorentzian half-widths α_G and α_L, respectively, as well $\upsilon-\upsilon_0$ that defines the centre of the Voigt line profile. This function is approximated to compute the Voigt function quickly and rationally [1]. The fitting algorithm is written in MATLAB as a nonlinear least-squares minimisation problem, with trust-region.

For spectral fits in the three-Voigt Model, the only constraints of the fitting are the widths:

Parameter under constraint	Lower Bound	Upper Bound
α_G	10 meV	30 meV
α_L	0.1 meV	10 meV

The justification of these values come from transient optical measurements on TMD. [2] [3]

Four starting parameters are specified for each emission state, the exciton (X0), charged exciton (CX) and the localised exciton (LX): The peak position, intensity, Gaussian and Lorentzian widths. These starting values are presented in the table below. Peak position for the exciton state is known to be around 2 eV for WS_2, the values of the charged and localised exciton states are taken based on experimentally obtained and theoretically predicted binding energies for the CX and LX states, which are approximately 30 meV and 75 meV respectively. As the spectral intensity varies across the map, due to photoluminescence inhomogeneity, the maximum numerical value of measured PL intensity across all positions on the map (M) was used as the reference for the intensity starting values. Lastly, the starting values of the width parameters were chosen to be the middle of the constraints shown above. Before applying the fitting,

Emission state	Peak Position	Intensity	α_G	α_L
X0	2 eV	$\mathrm{I_{X0}} = \mathrm{M}$	15 meV	5 meV
CX	1.97 eV	$\mathrm{I_{X0}} = \mathrm{M}$	15 meV	5 meV
LX	1.92 eV	$\mathrm{I_{X0}} = \mathrm{M}/4$	15 meV	5 meV

The fitting procedure is performed by MATLAB, using the nonlinear least-squared method, it was found that within 35 iterations, convergence was reached for all twelve parameters.

The above procedure is applied to all points on recorded PL maps, from which parameter maps, e.g. S_{CX} were extracted. In order to reduce computation time, pixels that do not correspond to possible WS_2 spectra were ignored by the fitting algorithm, this was achieved by appropriately thresholding the integrated intensity across the PL map prior to applying fitting procedures.

Next it is demonstrated that the above fitting procedure yields optimal results and is highly adaptable in spite of spectral inhomogeneity. The PL map from Figure 3 in the main manuscript will be used as the example. Due to the large number of spectral samples in the map, three co-ordinates will be chosen from the map with different brightness levels, to represent the range of possible spectral shapes and signal-to-noise ratios with the corresponding measured and fitted PL, as well as the residuals. Finally a histogram showing the residuals for all pixels will be shown.

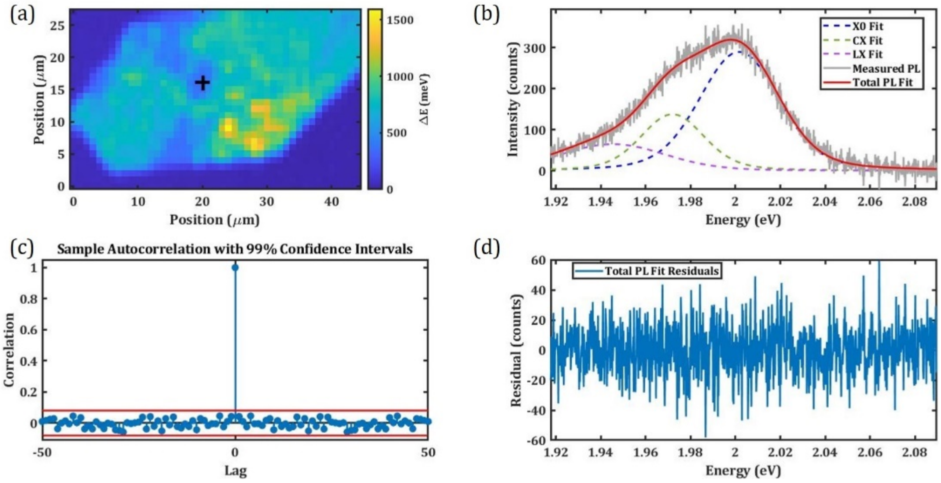

SM-Figure 1: Example of 3-Voigt model fitting the PL spectrum point (20,16) of the PL map shown in (a). The measured data fitted data are plotted in (b), the residuals in (d) and the autocorrelation of the residuals plotted in (c), which also shows the white noise autocorrelation upper and lower boundaries as red lines. The parameters obtained from this fit are: X0: $E = 2.00$ eV, $I = 369, \propto_G = 0.018, \propto_L = 0.004$, CX: $E = 1.98$ eV, $I = 186, \propto_G = 0.014, \propto_L = 0.005$, X0: $E = 1.95$ eV, $I = 77, \propto_G = 0.022, \propto_L = 0.004$.

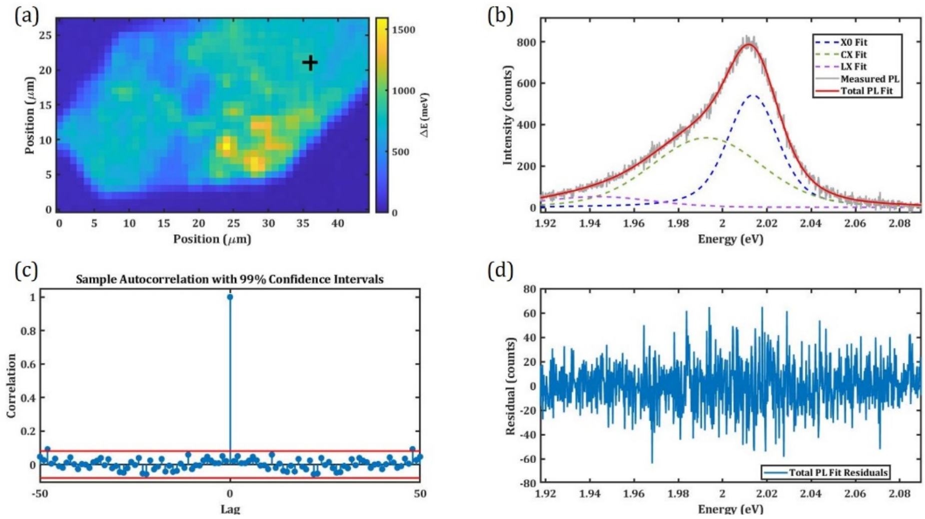

SM-Figure 2: Example of 3-Voigt model fitting the PL spectrum at point (36,21) of the PL map shown in (a). The measured data fitted data are plotted in (b), the residuals in (d) and the autocorrelation of the residuals plotted in (c), which also shows the white noise autocorrelation upper and lower boundaries as red lines. The parameters obtained from this fit are: X0: $E = 2.01$ eV, $I = 877, \propto_G = 0.01, \propto_L = 0.006$, CX: $E = 1.99$ eV, $I = 412, \propto_G = 0.026, \propto_L = 0.006$, X0: $E = 1.94$ eV, $I = 63, \propto_G = 0.026, \propto_L = 0.006$.

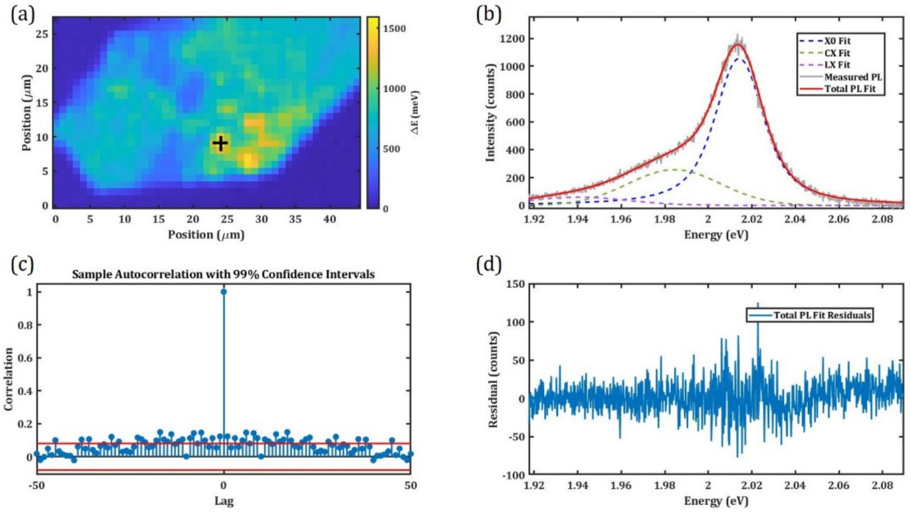

SM-Figure 3: Example of 3-Voigt model fitting the PL spectrum at point (24,9) of the PL map shown in (a). The measured data fitted data are plotted in (b), the residuals in (d) and the autocorrelation of the residuals plotted in (c), which also shows the white noise autocorrelation upper and lower boundaries as red lines. The parameters obtained from this fit are: X0: $E = 2.01$ eV, $I = 2493, \propto_G = 0.011, \propto_L = 0.009$, CX: $E = 1.98$ eV, $I = 257, \propto_{\mathrm{G}} = 0.015, \propto_{\mathrm{L}} = 0.002$, X0: $E = 1.94$ eV, $I = 67, \propto_G = 0.025, \propto_L = 0.003$.

All other spectral fits on this flake had similar residuals with the largest being shown in SM-Figure 3. The autocorrelation plots in each case show that the fit had accounted for most of the PL emission signal, with the remainder caused by white noise or shot noise. In SM-Figure 2 (top panel), where we plotted the residuals for each fit on the same graph, clearly the residuals are shot-noise limited and the fit cannot be improved further. The relative errors of all shot-noise limited spectral fits are quantified by dividing the residual by the measured intensity at each energy value. According to the histogram shown in SM-Figure 5 (Lower panel), 92 % of all shot-noise limited data have relative error 10 % or less. Shot noise can also be eliminated from the residual analysis, by normalising the residual from each spectral fit by the mean residual of that fit, after which the relative error are reduced to below 2%. We compared this with a three-Gaussian model and plotted the analysed residuals in SM-Figure 4. Residuals from the three-Gauss model is not entirely random with oscillatory trends, with a visibly larger range compared to the three-Voigt model. The relative errors for the shot-noise corrected and uncorrected cases also follow the same contrast.

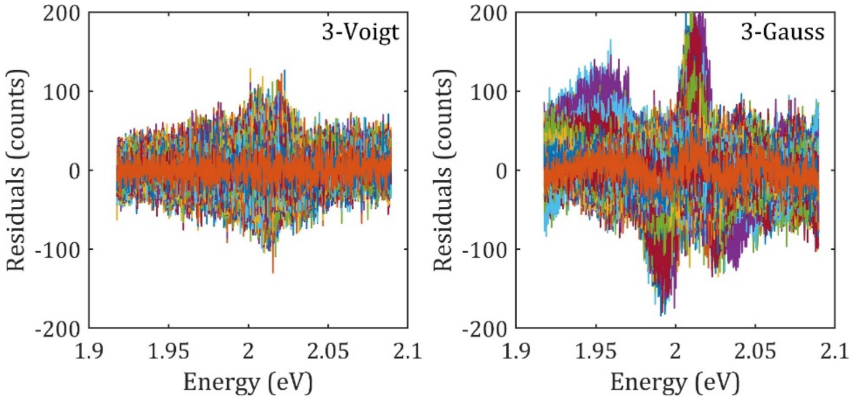

SM-Figure 4 Comparing the residuals from the entire PL map shown in SM-Figure 1,2 and 3 after fitting, with a 3-Voigt, against a 3-Gauss model.

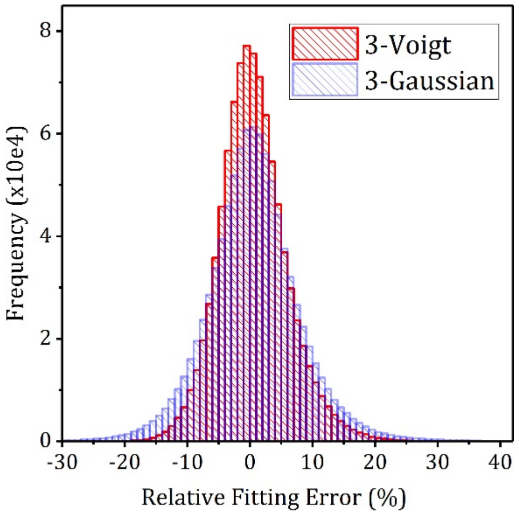

SM-Figure 5 Relative error histogram of all positions from the PL map, shown in SM-Figure 1,2 and 3 calculated from 3-Voigt model fitting and 3-Gauss model fitting.

2. Steady state model of the spectral weighting

The steady state model defined in Equations (1.1-1.5) returns the scattering parameters Γ_S^{CX} and Γ_S^{LX}, which are scattering rates describing the formation of charged excitons and localised exitons, respectively. The scattering rates obtained from the curve fitting are significantly slower compared to the values reported elsewhere [4], however studies that report much faster scattering rates, such as in MoS_2 also reported higher CX spectral weightings than X0. Here, the PL spectral characteristics imply $S_{X0} > S_{CX}, S_{LX}$, this could be the result of a shift in the dielectric constant of the PDMS substrate compared to the SiO_2/Si substrate.

3. Additional PL histogram for Figure 3

Figure 3 in the main text presents the illumination-time dependence for a small monolayer region studied using time-stepped PL maps. The histograms of the fitted PL intensity are not included in Figure 3, those data are presented here instead in SM-Figure 6 column (d), which shows that the mean intensity increases with illumination-time, tracking the corresponding changes in the S_{X0} and S_{CX}.

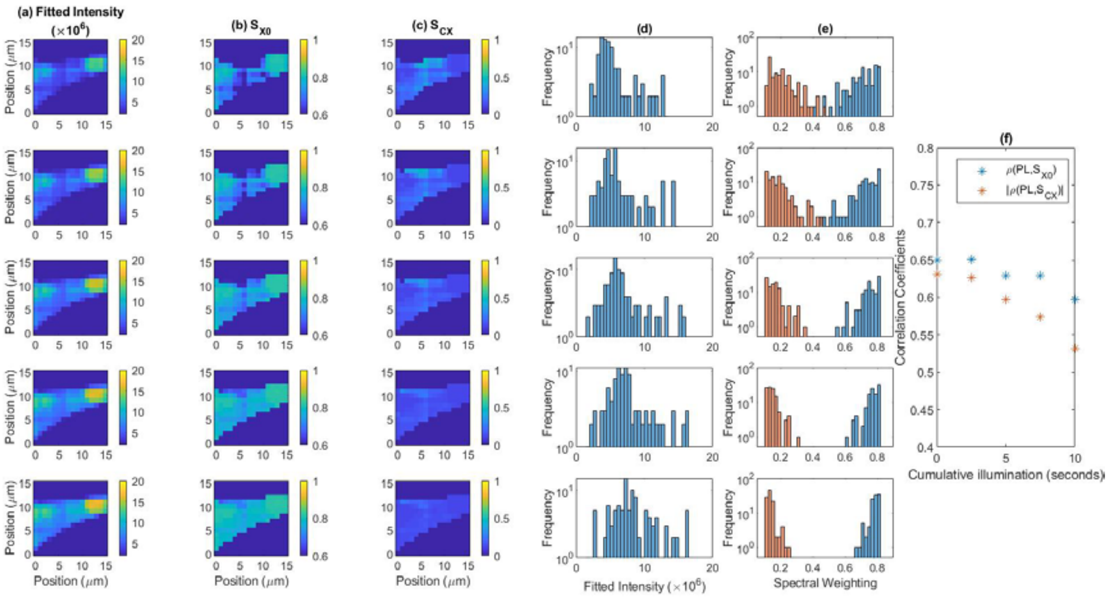

SM-Figure 6 Illumination-time dependence for a small monolayer region studied using time-stepped PL maps. (a) PL intensity and (b) S_{CX} values per $x-y$ position extracted at different laser illumination times. From top to bottom: $t = 0\ s$ (before laser illumination) and after repeated laser illumination time steps $t = 2.5\ s$, $5\ s$, $7.5\ s$ and $10\ s$. Histograms of S_{CX} values from each map are plotted in column (c). In (d), the fitted PL intensity histograms are plotted. In (e) histograms of S_{CX} and S_{X0} values at each stage is plotted. Correlation statistics between the PL intensity and each of the spectral weighting maps S_{CX} and S_{X0} are extracted and plotted against the laser illumination time in (f), with the absolute value taken for S_{CX}.

4. Changes to spectral homogeneity after 5 months.

After leaving the large flake in ambient conditions for 5 months after the final measurement, the spectral homogeneity was measured again. This data is presented here, serving to compare the spectral homogeneity of this flake before the laser illumination, immediately after laser illumination (Figure 5 from our manuscript), and then after several months with the monolayer/PDMS sample stored at ambient conditions.

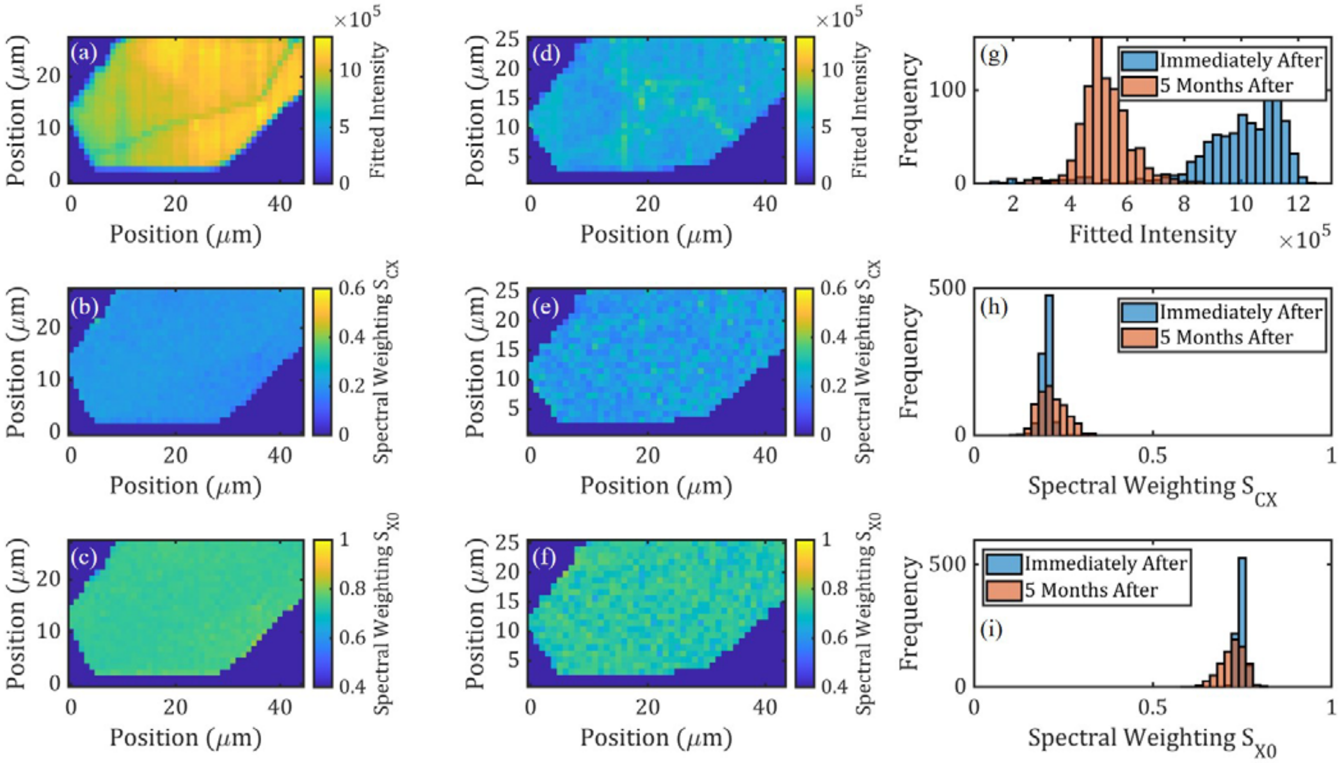

SM-Figure 7 Maps and statistics from large area WS_2 flake after laser illumination (a) and after 5 months (d), S_{CX} and S_{X0} maps were extracted from modelling the PL maps both immediately after (b)-(c) and 5 months after (e)-(f) the laser illumination, their spatial histograms are correspondingly plotted in (g) for the fitted intensity, in (h) for the charged exciton weighting and in (i) for the exciton spectral weightings.

We would like to emphasise that the laser irradiation does two things, 1. It reduces the mean spectral weighting, which means that the charged exciton emission dominance is reduced across all measured positions on the flake, and 2. It reduces the variance of the spectral weighting, which means that spectrally the flake's homogeneity has increased. After several months in the lab, stored at ambient conditions, we see that only the variance is "recovering" to its initially broad distribution, whereas the mean has stayed approximately unchanged. This indicates that the recovery is only partial. The recoverable component is likely to be due to contaminants physisorbed onto the monolayer during its storage time, whereas the non-recoverable component is indicative of a permanent passivation of structural or chemical defects.

[1] S. M. Abrarov and B. M. Quine, J. Math. Res. 7, (2015).

[2] M. Selig, G. Berghäuser, A. Raja, P. Nagler, C. Schüller, T. F. Heinz, T. Korn, A. Chernikov, E. Malic, and A. Knorr, Nat. Commun. 7, 13279 (2016).

[3] O. A. Ajayi, J. V. Ardelean, G. D. Shepard, J. Wang, A. Antony, T. Taniguchi, K. Watanabe, T. F. Heinz, S. Strauf, X.-Y. Zhu, and J. C. Hone, 2D Mater. 4, 031011 (2017).

[4] H. Shi, R. Yan, S. Bertolazzi, J. Brivio, B. Gao, A. Kis, D. Jena, H. G. Xing, and L. Huang, ACS Nano 7, 1072 (2013).
